# Supplementary material for: Development of a Method for the In Vivo Generation of Allogeneic Hearts in Chimeric Mouse Embryos
Source: Int J Mol Sci. 2023 Jan 6;24(2):1163. doi: 10.3390/ijms24021163 (PMC9865658; doi:10.3390/ijms24021163)
Supplement: Supplementary file 1 [file ijms-24-01163-s001.zip › ijms-2043355-supplementary.pdf]

Supplementary Material

Figure S1. CRISPR Off-Target analysis.

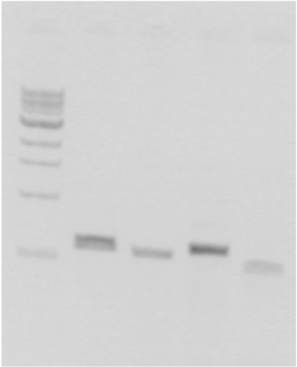

Gel image showing genomic PCR to isolate off-target sequences. Primer sequences are listed in Supplementary Table S2.
Lane 1: 1Kb DNA ladder, Lane 2: Off-Target 1, Lane 2: Off-Target 2, Lane 4: Off-Target 3, Lane 5: Off-Target 13.

Off-Target 1

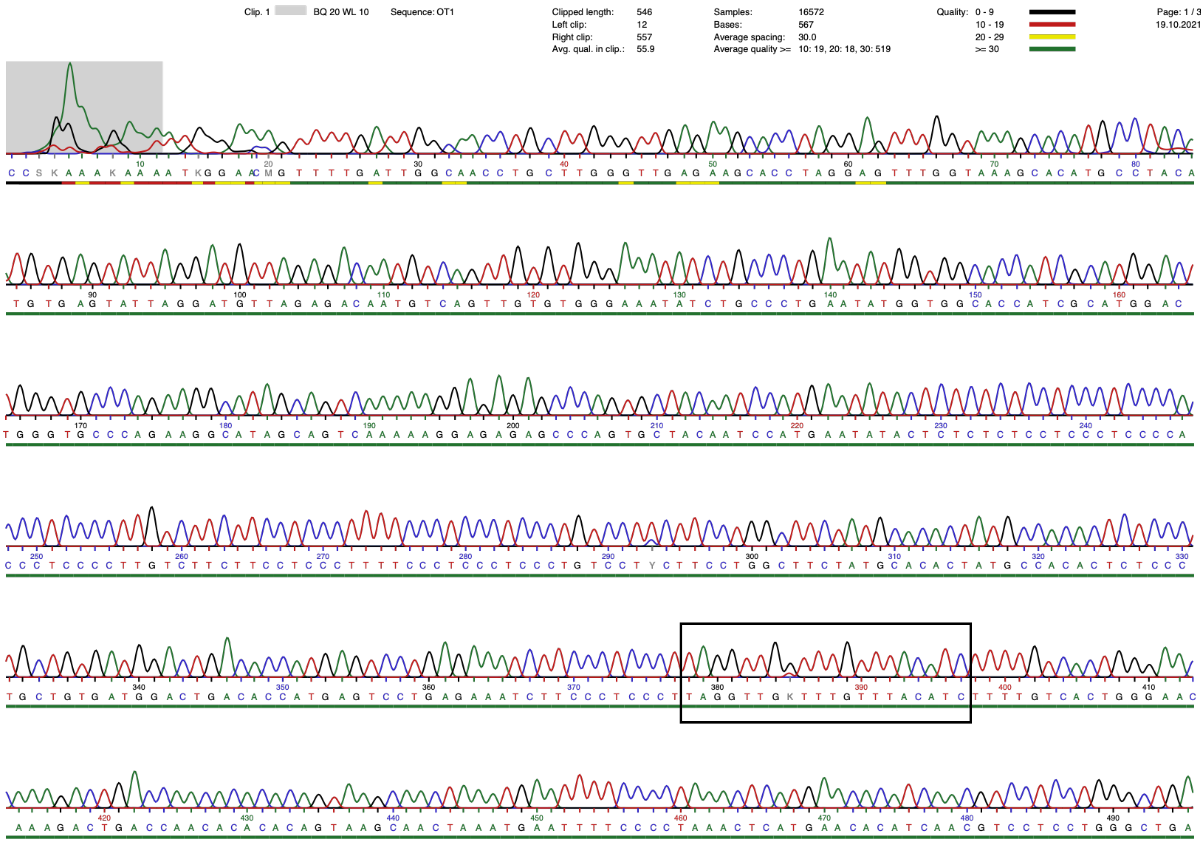

Sequencing results for Off-Target 1 on Chr14. Framed is the sequence that can be recognized by Cas9.

```
Query  224       CCCAccctccccttgtcttcttcctccctttt ccctccctccctgtcctycttcctGGCT  283
                 ||||||||||||||||||||||||||||||||||||||||||||||||| ||||||||||
Sbjct  77196196  CCCACCCTCCCCTTGTCTTCTTCCTCCCTTTTCCCTCCCTCCCTGTCCTCCTTCCTGGCT  77196255

Query  284       TCTATGCACACTATGCCACACTCTCCCTGCTGTGATGGACTGACACCATGAGTCCTGAGA  343
                 ||||||||||||||||||||||||||||||||||||||||||||||||||||||||||||
Sbjct  77196256  TCTATGCACACTATGCCACACTCTCCCTGCTGTGATGGACTGACACCATGAGTCCTGAGA  77196315

Query  344       AATCTTCCCTCCCTTAGGTTGKTTTGTTTACATCTTTTGTCACTGGGAACAAAGACTGAC  403
                 |||||||||||||||||||| |||||||||||||||||||||||||||||||||||||||
Sbjct  77196316  AATCTTCCCTCCCTTAGGTTGTTTTGTTTACATCTTTTGTCACTGGGAACAAAGACTGAC  77196375

Query  404       CAACACACACAGTAAGCAACTAAATGAATTTTCCCCTAAACTCATGAACACATCAACGTC  463
                 ||||||||||||||||||||||||||||||||||||||||||||||||||||||||||||
Sbjct  77196376  CAACACACACAGTAAGCAACTAAATGAATTTTCCCCTAAACTCATGAACACATCAACGTC  77196435

Query  464       CTCCTGGGCTGAGTACCAGCGAAACTCAAAAGGGAAGCATGCGCTCTTGAATCATAAGTG  523
                 ||||||||||||||||||||||||||||||||||||||||||||||||||||||||||||
Sbjct  77196436  CTCCTGGGCTGAGTACCAGCGAAACTCAAAAGGGAAGCATGCGCTCTTGAATCATAAGTG  77196495

Query  524       TGGAAAT  530
                 |||||||
Sbjct  77196496  TGGAAAT  77196502
```

Alignment of Off-Target 1 sequencing results with genomic DNA. Framed is the sequence that can be recognized by Cas9. The two sequences are identical. K is a known polymorphism.

Off-Target 2

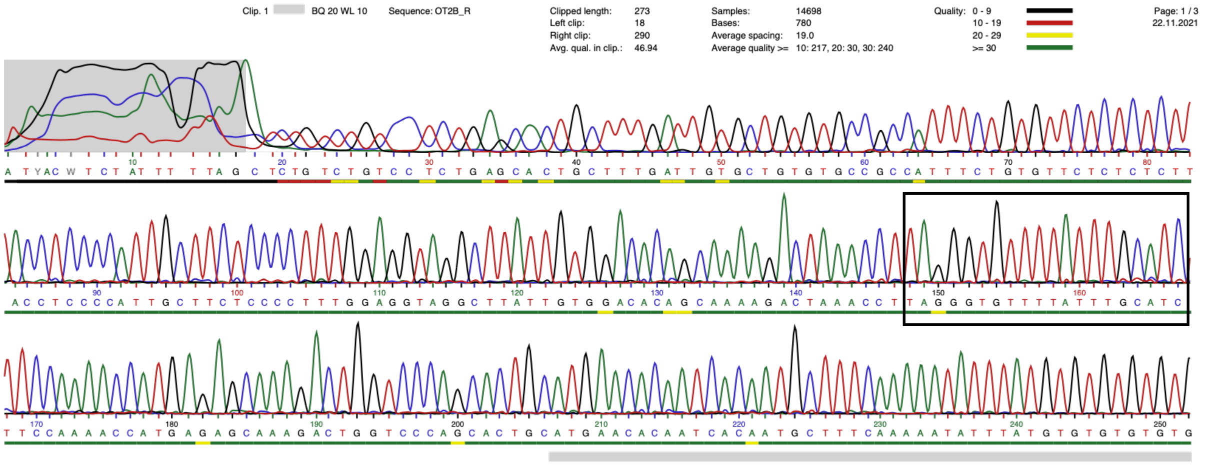

Sequencing results for Off-Target 2 on Chr16. Framed is the sequence that can be recognized by Cas9.

```
Query  2         CTGTCTGT-CCTCTGAGC-ACTGCTTTGATTGTGCTGTGTGCCGCCATTTCTGTGTTCTC  59
                 |||||||| ||||||||| ||||||||||||||||||||||||||||||||||||||||||
Sbjct  91342498  CTGTCTGTCCCTCTGAGCAACTGCTTTGATTGTGCTGTGTGCCGCCATTTCTGTGTTCTC  91342439

Query  60        TCTCTTACCTCCCCATTGCTTCTCCCCTTTGGAGGTAGGCTTATTGTGGACACAGCAAAA  119
                 ||||||||||||||||||||||||||||||||||||||||||||||||||||||||||||
Sbjct  91342438  TCTCTTACCTCCCCATTGCTTCTCCCCTTTGGAGGTAGGCTTATTGTGGACACAGCAAAA  91342379

Query  120       GACTAAACCTTAGGGTGTTTTATTTGCATCTTCCAAAACCATGAGAGCAAAGACTGGTCC  179
                 ||||||||||||||||||||||||||||||||||||||||||||||||||||||||||||
Sbjct  91342378  GACTAAACCTTAGGGTGTTTTATTTGCATCTTCCAAAACCATGAGAGCAAAGACTGGTCC  91342319

Query  180       CAGCACTGCATGAACACAATCACAATGCTTTCAAAAATATTTa  222
                 |||||||||||||||||||||||||||||||||||||||||||
Sbjct  91342318  CAGCACTGCATGAACACAATCACAATGCTTTCAAAAATATTTA  91342276
```

Alignment of Off-Target 2 sequencing results with genomic DNA. Framed is the sequence that can be recognized by Cas9. The two sequences are identical.

Off-Target 3

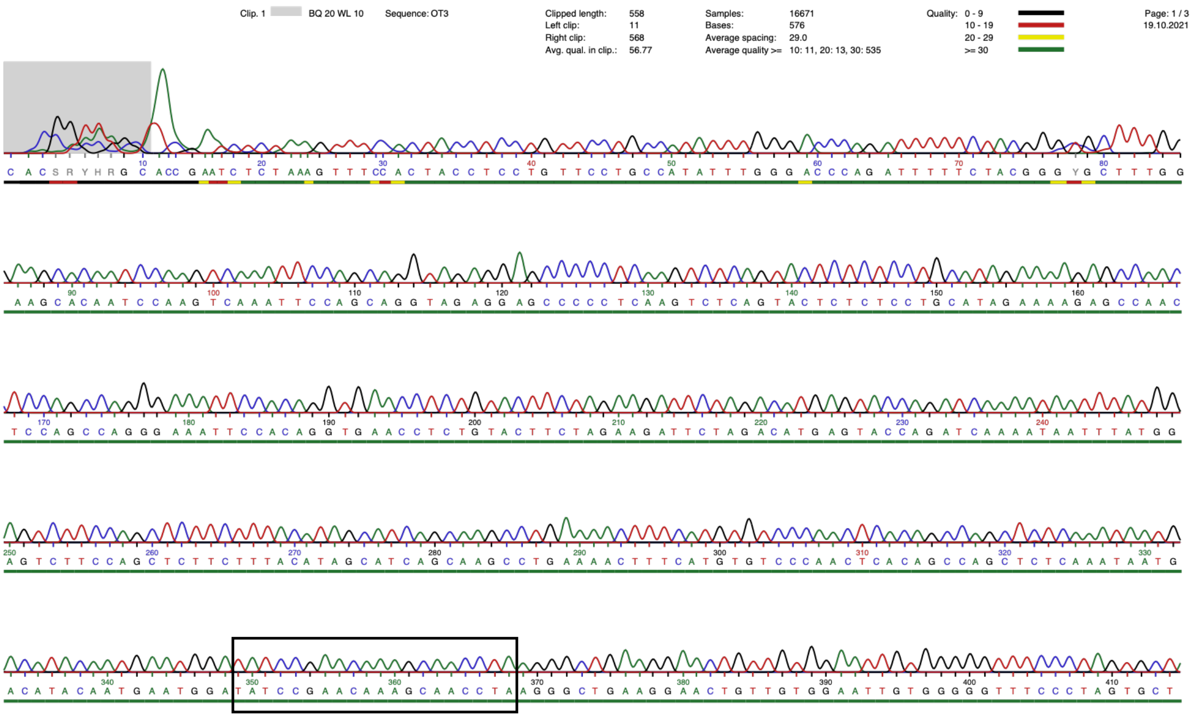

Sequencing results for Off-Target 3 on Chr1. Framed is the sequence that can be recognized by Cas9.

```
Query  181       GTACTTCTAGAAGATTCTAGACATGAGTACCAGATCAAAATAATTTATGGAGTCTTCCAG  240
                 ||||||||||||||||||||||||||||||||||||||||||||||||||||||||||||
Sbjct  42684230  GTACTTCTAGAAGATTCTAGACATGAGTACCAGATCAAAATAATTTATGGAGTCTTCCAG  42684289

Query  241       CTCTTCTTTACATAGCATCAGCAAGCCTGAAAACTTTCATGTGTCCCAACTCACAGCCAG  300
                 ||||||||||||||||||||||||||||||||||||||||||||||||||||||||||||
Sbjct  42684290  CTCTTCTTTACATAGCATCAGCAAGCCTGAAAACTTTCATGTGTCCCAACTCACAGCCAG  42684349

Query  301       CTCTCAAATAATGACATACAATGAATGGATATCCGAACAAAGCAACCTAAGGGCTGAAGG  360
                 || ||||||||||||||||||||||||||||||||||||||||||||||| ||||||||||
Sbjct  42684350  CTTTCAAATAATGACATACAATGAATGGATATCCGAACAAAGCAACCTAAGGGCTGAAGG  42684409

Query  361       AACTGTTGTGGAATTGTGGGGGTTTCCCTAGTGCTAGTAATCAAACAAAGRAACTCACAC  420
                 |||||||||||||||||||||||||||||||||||||||||||||||||| |||||||||
Sbjct  42684410  AACTGTTGTGGAATTGTGGGGGTTTCCCTAGTGCTAGTAATCAAACAAAGAAACTCACAC  42684469

Query  421       TTGCTAGACAAGGTGTTCTACTGCTGAGCAGCCCAGCTAAGTACAGGACCTTTCTAAAAG  480
                 ||||||||||||||||||||||||||||||||||||||||||||||||||||||||||||
Sbjct  42684470  TTGCTAGACAAGGTGTTCTACTGCTGAGCAGCCCAGCTAAGTACAGGACCTTTCTAAAAG  42684529

Query  481       ACACATGATGTAAACAAACAGCATATTAGAAAGAACGGCAACATAAAGTCTAA  533
                 |||||||||||||||||||||||||||||||||||||||||||||||||||||
Sbjct  42684530  ACACATGATGTAAACAAACAGCATATTAGAAAGAACGGCAACATAAAGTCTAA  42684582
```

Alignment of Off-Target 3 sequencing results with genomic DNA. Framed is the sequence that can be recognized by Cas9. The two sequences are identical.

Off-Target 13

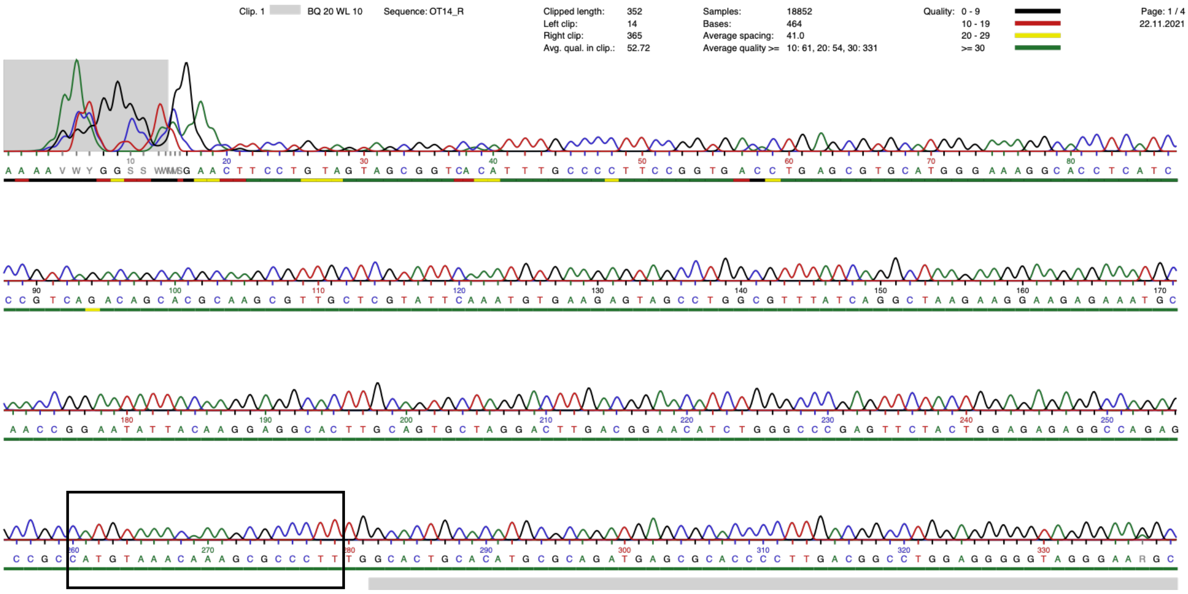

Sequencing results for Off-Target 13 in *Ncapd3*. Framed is the sequence that can be recognized by Cas9.

```
Query  3         ACTTCCTGTAGTAGCGGTCACATTTGCCCCTTCCGGTGAC-CTGAGCGTGCATGGGAAAG  61
                 |||||||||||||||||||||||||||||||||||||||| |||||||||||||||||||
Sbjct  26941743  ACTTCCTGTAGTAGCGGTCACATTTGCCCCTTCCGGTGACACTGAGCGTGCATGGGAAAG  26941684

Query  62        GCACCTCATCCCGTCAGACAGCACGCAAGCGTTGCTCGTATTCAAATGTGAAGAGTAGCC  121
                 ||||||||||||||||||||||||||||||||||||||||||||||||||||||||||||
Sbjct  26941683  GCACCTCATCCCGTCAGACAGCACGCAAGCGTTGCTCGTATTCAAATGTGAAGAGTAGCC  26941624

Query  122       TGGCGTTTATCAGGCTAAGAAGGAAGAGAAATGCAACCGGAATATTACAAGGAGGCACTT  181
                 ||||||||||||||||||||||||||||||||||||||||||||||||||||||||||||
Sbjct  26941623  TGGCGTTTATCAGGCTAAGAAGGAAGAGAAATGCAACCGGAATATTACAAGGAGGCACTT  26941564

Query  182       GCAGTGCTAGGACTTGACGGAACATCTGGGCCCGAGTTCTACTGGAGAGAGGCCAGAGCC  241
                 ||||||||||||||||||||||||||||||||||||||||||||||||||||||||||||
Sbjct  26941563  GCAGTGCTAGGACTTGACGGAACATCTGGGCCCGAGTTCTACTGGAGAGAGGCCAGAGCC  26941504

Query  242       GCCATGTAAACAAAGCGCCCTTTGGCACTGCACATGCGCAGATGAGCGCACCCCTTGACG  301
                 ||||||||||||||||||||||||||||||||||||||||||||||||||||||||||||
Sbjct  26941503  GCCATGTAAACAAAGCGCCCTTTGGCACTGCACATGCGCAGATGAGCGCACCCCTTGACG  26941444

Query  302       GCCTGGAGGGGGTAGGGAARGCGAgggggggggg  335
                 ||||||||||||||||||| ||||||||||||||
Sbjct  26941443  GCCTGGAGGGGGTAGGGAAAGCGAGGGGGGGGGG  26941410
```

Alignment of Off-Target 13 sequencing results with genomic DNA. Framed is the sequence that can be recognized by Cas9. The two sequences are identical.

Table S1. Primers used to check insertion of transgenes.

**ext1-F**	**TGGATCTGGGGCCATAAATGCT**
**int1-R**	**ACCGGACACTTCCCTATGTG**
**int2-R**	**TGTGCTTGGTCACTGCAGAG**

Table S2. Primers used in the Off-Target analysis.

	**Forward (5′-3′)**	**Reverse (5′-3′)**
**Off-Target 1**	**GAAAGATGTCTCCGAGAGAGGG**	**CGCAGCTCGAATTTCCACACTTA**
**Off-Target 2**	**ACCGGACACTTCCCTATGTG**	**TGGCAGCGGAAGATAACAGG**
**Off-Target 3**	**CCTCGGTGGCATACCTTCAA**	**AGGTGTAAAGGGTGGGTCT**
**Off-Target 13**	**CGTAACGCACCGAAGTGCTA**	**TCTTACACGGCAGGGAACCT**

Table S3. Primers used in Real-Time RT-PCR analysis.

	**Forward (5′-3′)**	**Reverse (5′-3′)**
**GADPH**	**AGGTCGGTGTGAACGGATTTG**	**TGTAGACCATGTAGTTGAGGTCA**
**Nkx2.5**	**CTATGCCCTGTCCCTCAGAT**	**CTCCCGGTCCTAGTGTGGAA**
**GFP**	**GAAGCAGCACGACTTCTTCAA**	**AAGTCGATGCCCTTCAGCTC**
